# Developmental and epileptic encephalopathy with spike–wave activation in sleep: From the ‘functional ablation’ model to a neurodevelopmental network perspective

**DOI:** 10.1111/dmcn.16361

**Published:** 2025-05-12

**Authors:** Luca Andreoli, Stefania Maria Bova, Pierangelo Veggiotti

**Affiliations:** ^1^ Neuroscience Research Centre, Department of Biomedical and Clinical Sciences University of Milan Milan Italy; ^2^ Paediatric Neurology Unit Buzzi Children's Hospital Milan Italy

## Abstract

The interplay between epilepsy and cognition is intricate and multifaceted, particularly in the context of childhood‐onset epileptic disorders where epileptic activity can significantly interfere with and disrupt the delicate, highly plastic, and environment‐related trajectories of neurodevelopment. Developmental and epileptic encephalopathy with spike–wave activation during slow sleep (D/EE‐SWAS), a spectrum of conditions including Landau‐Kleffner syndrome, could serve as a valuable model to explore these complexities. Research to date has primarily examined its distinctive features, including genetic and structural etiological factors, electroencephalographic patterns, and cognitive phenotypes, often interpreted through simplified cause–effect paradigms. The adoption of a network perspective that aligns with neurodevelopmental trajectories is essential to grasp the full complexity of this evolving condition. Advancing research requires the integration of multimodal data, while leveraging tools such as artificial intelligence to develop sophisticated models in order to achieve a holistic understanding of D/EE‐SWAS.

## Graphical abstract

The pathophysiology of D/EE‐SWAS has traditionally been conceptualized as a cause–effect continuum wherein genetic and environmental factors influence brain morphology, subsequently inducing electrical abnormalities during sleep, resulting in cognitive impairment. A network‐based perspective could play a key role towards a comprehensive understanding of this complex condition, allowing the reframing of each individual element in its dynamic interactions with neurodevelopmental processes.

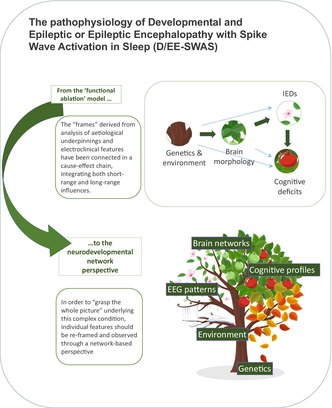

Plain language summary: https://onlinelibrary.wiley.com/doi/10.1111/dmcn.16374

**Abbreviations**
D/EE‐SWAS: developmental and epileptic encephalopathy with spike–wave activation in sleep
DMN: default mode network
EE‐SWAS: epileptic encephalopathy with spike–wave activation in sleep
fMRI: functional magnetic resonance imaging

**What this paper adds**

- The ‘functional ablation’ model cannot exhaustively account for cognitive features in developmental and epileptic encephalopathy with spike–wave activation during slow sleep (D/EE‐SWAS).
- A neurodevelopmental network perspective could provide a framework to comprehend D/EE‐SWAS.

Epileptic encephalopathy with spike–wave activation in sleep (EE‐SWAS) and developmental and epileptic encephalopathy with spike–wave activation in sleep (DEE‐SWAS), collectively known as D/EE‐SWAS, are a spectrum of rare childhood‐onset epilepsies featuring marked activation of spike–waves during sleep in association with stagnation or deterioration in cognitive, language, behavioural, and/or motor skills.^1^

Since cognitive plateauing or regression is a key feature of the condition in most patients, D/EE‐SWAS seems an appropriate model and a unique framework for exploring the complex interplay between epilepsy and cognition in childhood, when epileptic activity can significantly interfere with the delicate and highly plastic neurodevelopmental trajectories.

The potential link between abnormal electrical activity and cognitive alterations was seminally identified in 1942 by Kennedy and Hill in a child with cognitive decline and slow spike‐and‐wave electroencephalographic (EEG) abnormalities without overt seizures (incisively defined ‘dementia dysrythmica infantum’).^2^ In 1957 Landau and Kleffner described five children with acquired epileptic aphasia and diffuse interictal epileptiform discharges, prominently localized on temporal regions.^3^ Describing the syndrome, hence called Landau–Kleffner syndrome, they pointed out a possible correlation between the severity of clinical features and interictal epileptiform discharges. The importance of sleep in potentiating EEG abnormalities in children with clinically evident cognitive regression was then demonstrated by Patry, Lyagoubi, and Tassinari in 1971 who defined the ensuing clinical condition as electrical status epilepticus during slow sleep.^4^ Subsequent studies of large case series supported the concept of a cause–effect relationship between interictal epileptiform discharges, sleep deregulation, and cognitive regression and yielded an ever‐growing knowledge about the ‘encephalopathic’ nature of this condition associating spike–wave anomalies markedly active in non‐rapid eye movement sleep with cognitive/behavioural regression and heterogeneous seizures. The literature has increasingly been characterized by a degree of ambiguity due to the proliferation of nosological terms, such as electrical status epilepticus during sleep and continuous spike–waves during slow‐wave sleep, as well as a lack of clarity regarding whether these terms referred to the clinical condition itself or merely to the associated EEG pattern.^5^ In contrast, the latest International League Against Epilepsy definition provides greater precision by defining SWAS as an EEG pattern that can not per se constitute a diagnosis in the absence of cognitive regression or plateauing.

Against this background, the focus of debate and critical inquiry has remained over the complex interplay between electrical abnormalities and clinical deterioration, which can be framed either as independent epiphenomena of an underlying disorder or as strictly bonded in a causal chain giving rise to an epileptic encephalopathy. Indeed, the latter has been overshadowed since Landau and Kleffner speculated about a ‘functional ablation’ of cortical areas involved in language induced by persistent convulsive discharges.^3^ The iconic definition of ‘Penelope syndrome’ issued by Tassinari et al. reinforced this causal correlation and placed sleep in the foreground, leveraging on the analogy between sleep‐activated spike–waves and the mythical wife of Odysseus, both undoing overnight what has been spun during the day.^6^ Despite its clarity and straightforwardness, this pathogenetic model cannot fully explain the clinical variability ranging from uncoupled electroclinical fluctuations in the evolutions of single cases, to individuals harbouring marked spike‐wave activation in sleep without any clinical sign of deterioration.

Although some authors argued that it represents a step back as compared to other terminologies which hinged on the ‘epileptic encephalopathy’ concept (e.g. electrical status epilepticus during slow sleep, Penelope syndrome),^7^ the current definition by the International League Against Epilepsy may nonetheless open up new avenues for understanding and research. On one hand, the D/EE‐SWAS concept highlights the critical importance of accurately characterizing developmental trajectories both before and after the onset of SWAS, encouraging clinicians to consider this condition from a developmental perspective, rather than merely as an acquired acute‐onset disease. On the other hand, the new nosological definition departs from the view of a direct electroclinical cause–effect relationship, refraining from taking a definitive stance on the matter, and merely grounding the syndrome on the temporal association between electrical and clinical features. Furthermore, less stringent criteria regarding EEG pattern places more emphasis on diagnostic assessment and monitoring of cognitive and behavioural features, given the manifest relevance of neuropsychological long‐term outcome.^8^ In this respect, lack of thorough characterization of neuropsychological and behavioural profiles, including accurate description of psychiatric diagnoses, has been recently pointed out as a limitation of case series and drug trials involving patients with D/EE‐SWAS.^9^

## ‘CLASSICAL’ NEUROPSYCHOLOGICAL SYNDROMES ASSOCIATED WITH SWAS

Since the first descriptions, great effort has been devoted to producing detailed and representative characterizations of clinical cases featuring impairments in specific cognitive domains, with an eye on possible correlations between neuropsychological profiles and EEG focus, reflecting a deeper understanding of the complex interplay between brain structure and function.^10^

These prototypical presentations reflect a localizationist and modularist perspective that posits that cognitive functions are localized within brain modules, each responsible for specific cognitive processes and behaviours, a conceptual framework that has served as a crucial instrument in advancing the neuropsychology of epilepsy. However, emerging evidence has increasingly challenged the notion of a direct correlation between cortical areas and cognitive functions, while evolving conceptual frameworks now favour network‐based models, highlighting widespread network dysfunction as the primary driver of cognitive impairment.

In the context of SWAS‐related neuropsychological syndromes, Landau–Kleffner syndrome is characterized by temporal discharges, which have been linked to language dysfunction.^3^ In spite of this alleged specificity, concomitant deficit in visuomotor integration, visuospatial perception, and memory have been consistently reported, as well as behavioural disorders of various types.^10^ Cerebral visual impairment, particularly deficits in object recognition and Kanji dysgraphia, has been described in patients with occipito‐temporal SWAS, pointing to major impairment of the ventral stream.^11^ Frontal and generalized EEG abnormalities have been related to global regression with behavioural and executive impairments defining an acquired epileptic dementia, purportedly due to prefrontal involvement. Hallmark features include mood fluctuations and severe behavioural impairment, with attention deficit and hyperactivity and variable intellectual deterioration, impairment of higher verbal and non‐verbal reasoning skills as opposed to preservation of basic perceptive, spatial, and linguistic abilities.^12^ Psychotic features have been described in the context of significant cognitive and behavioural regression in patients who also showed transient but complete loss of language.^10^ Such pervasive regressions, leading to loss of communicative competences and behavioural regulation, have also been framed as specimens of autism spectrum disorder‐like regression, raising questions on the interplay between epilepsy and autism.^13^ The adoption of a psychiatric point of view and taxonomy, in lieu of a neuropsychological perspective, could yield different diagnostic interpretations for these behavioural disorders.

Notwithstanding their historical significance and representativeness, these classical lesion‐based profiles inadequately account for substantial clinical heterogeneity. Prototypical neuropsychological syndromes related to SWAS are seldom seen in clinical practice, where patients display diverse mixtures of symptoms affecting multiple neuropsychological and behavioural domains to varying degrees. Contrasting evidence has been produced about correlations between EEG focus and specific symptoms, arguing against definite localization‐related neuropsychological syndromes.^14^ Nonetheless, current neuropsychological phenotyping in D/EE‐SWAS remains predominantly reliant on these traditional prototypes, as well as on neurodevelopmental syndromes (e.g. autism spectrum disorder, attention‐deficit/hyperactivity disorder), and on the use of Full‐scale IQ and other compound cognitive scores.^15^ These accounts run the risk of oversimplification, potentially failing to capture the complexity of the neurobehavioural impairment associated with D/EE‐SWAS.

## A NEW FRAMEWORK TO ADDRESS COMPLEXITIES

D/EE‐SWAS emerges as an inherently complex condition, and understanding complexities requires advanced methods for analysis and interpretation. Among the most promising tools emerging over the last decades, computational modelling has catalysed a paradigm shift towards an integrative approach of brain functioning through the lens of graph theory.^16^ This breakthrough view, referred to as the network or connectome perspective, emphasizes the brain's complex web of interconnected regions whose dynamic interactions underlie macroscale functions, enhancing our understanding of the relationship between brain activity and behaviour. The application of graph theory allows for the exploration of brain networks and their hierarchical organization, providing different centrality measures. Network hierarchy is determined by the reciprocal configuration of hubs—key nodes playing a relay function thanks to their connectivity and their strategic location at the intersection of significant fibre pathways for efficient network communication—and their interconnections form the basis of brain functioning. This ‘small‐world topology’, characterized by highly clustered subnetworks, promotes both modular segregation and integration by enabling widely distributed yet interconnected information processing in a hierarchical mode. At the highest level, a distinct set of densely interconnected core hubs forms an exclusive group known as ‘rich club’, acting as the primary conductors for signal transmission and integrating inputs from more isolated nodes with hyperspecialized functional roles.

Key features of brain networks are thus represented by a genetically determined hierarchical structure which combines with elevated age‐specific plasticity and significant adaptability to internal and external influences, giving rise to highly specialized functions that are optimally aligned with environmental demands. During brain development, networks are structured through continuous interaction between genetic and environmental factors, following defined trajectories from small‐scale to larger integrated systems within critical developmental windows.^17^ Emerging seminal insights are elucidating these developmental timelines from early visual and sensorimotor through higher order networks.^18^

Brain networks and hubs can be mapped using non‐invasive methods, especially neuroimaging and neurophysiological techniques that allow investigation of in vivo structural and functional connectivity. Structural connectivity refers to anatomical pathways linking distinct brain regions and it can be documented with advanced neuroimaging techniques, including diffusion magnetic resonance imaging (MRI) tractography and covariance analysis of morphological MRI markers. Conversely, functional connectivity refers to the temporal correlations between activity patterns in different brain regions, and it can be investigated using functional magnetic resonance imaging (fMRI) or EEG/magnetoencephalography, providing insights into dynamic network interactions during both resting‐state and task‐related conditions.

Although graph theory has primarily been applied to the study of the brain's physiological functions and more recently on neurodevelopmental disorders, this perspective could also be pivotal in advancing our understanding of the neurocognitive aspects of epilepsy syndromes, allowing correlations between network connectivity metrics and quantitative measures of cognitive functions. Indeed, this approach aligns with the Research Domain Criteria framework, which advocates for a neurobiological and network‐based view of cognitive disorders and promotes the study of neurobehavioural domains through dimensions of functioning rather than mere diagnostic labelling.^19^

From a network perspective, epilepsy has similarly been framed as resulting from the dysfunction of specific neural systems.^20^ Recent advances in the field have increasingly emphasized the critical involvement of hub regions, which exhibit structural and functional alterations potentially linked to atypical neural reorganization, as observed in childhood epilepsy syndromes.^21^, ^22^

**FIGURE 1:**
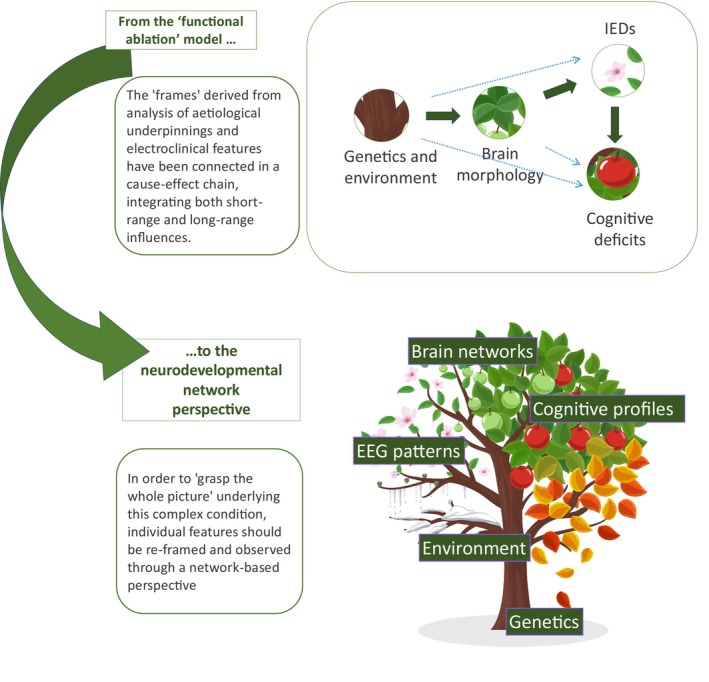
The pathophysiology of developmental and epileptic encephalopathy with spike‐wave activation in sleep (D/EE‐SWAS) has been investigated over the years through an in‐depth analysis of its aetiological underpinnings and electroclinical features. The emerging ‘frames’, here metaphorically represented as components of a tree (trunk, leaves, flowers, and fruits), have been connected in a cause–effect chain, integrating both short‐range and long‐range influences. This ‘classical’ pathophysiological cascade begins with the interplay between genetic and environmental factors, which shape brain morphology. These macro‐ and microstructural alterations subsequently contribute to the activation of interictal epileptiform discharges during sleep, ultimately leading to cognitive decline. In this framework, each element contributes to the global system through multidirectional dynamics that unfold over time, highlighting the dynamic and interconnected nature of the disorder's pathophysiology. Abbreviations: EEG, electroencephalogram; IED, interictal epileptiform discharge

To date, research on D/EE‐SWAS has primarily focused on its distinctive features, including genetic and structural etiological factors, EEG patterns, and cognitive phenotypes, often tentatively connected through direct cause–effect relationships. Embracing a network perspective rooted in neurodevelopmental trajectories may be essential for capturing the inherent complexity of this condition as it evolves over time, thereby providing a more comprehensive point of view to ‘grasp the whole picture’ (Figure 1).

## EVIDENCE IN D/EE‐SWAS FROM A NETWORK PERSPECTIVE

D/EE‐SWAS can be regarded as a sophisticated yet powerful model that offers a framework for understanding how brain networks are shaped during developmental phases. In particular, this model could highlight the interplay of structural and functional noxae, as well as genetic and environmental influences, which can modify developmental trajectories at various critical periods by affecting the maturation and organization of brain networks.

A substantial body of historical and widely accepted evidence suggests that network theory may provide a viable framework for the comprehension of D/EE‐SWAS through the lens of neurodevelopment. Indeed, this condition features a strong and age‐dependent temporal connection with the development of brain networks subserving high‐order functions such as language and behaviour.^23^ The perisylvian network has been indicated as a critical component in the pathophysiology of D/EE‐SWAS, with supporting evidence from source reconstruction analysis.^24^ These findings have provided grounds for theories positing that D/EE‐SWAS exists on a spectrum continuum with self‐limited focal epilepsies, collectively conceptualized as a developmental disorder of the perisylvian network.^25^ Evidence concerning genetic underpinnings corroborates this strong association, given the involvement of genes that are differentially expressed during subsequent neurodevelopmental phases. Proposed correlations between gene variants and average age of D/EE‐SWAS onset could further substantiate this hypothesis, although additional evidence is required.^26^ Recent research has identified two major genetic clusters (encoding for ionic channels and transcriptional regulators) whose disruption is linked to both DEE‐SWAS and EE‐SWAS.^27^ This DEE‐EE continuum could reflect shared underlying mechanisms that drive maladaptive interactions between neurodevelopmental and epileptogenic processes, which together shape genetically determined brain networks through their epigenetic vulnerability to environmental stimuli. Lastly, from a clinical viewpoint the onset and natural resolution of SWAS are closely tied to critical periods during which the brain demonstrates heightened plasticity and vulnerability to external stimuli that shape its structural and functional maturation. This temporal intersection can lead to dysfunctional plasticity within cortico‐cortical and thalamocortical circuits, particularly through the disruption of non‐rapid eye movement slow‐wave activity, which is vital for synaptic homeostasis and network stability. Indeed, in analogy with other childhood epilepsies, the localization of epileptic foci in D/EE‐SWAS exhibits an age‐related posterior–anterior progression, paralleling maximal cortical thickness and slow‐wave activity, thereby suggesting a close association with brain maturation. Moreover, when cortical structures are thus shaped by aberrant electrical activity during these critical periods, they may become misaligned with the actual sensory environment. When effectively ‘cemented’ at the end of the developmental window of opportunity, these maladapted structures can result in long‐term impairments.

In recent years, advanced neuroimaging and neurophysiological techniques have been employed to characterize brain networks in D/EE‐SWAS and to elucidate disruptions in functional connectivity, providing valuable insights into the underlying pathomechanisms.^28^

Thalamo‐cortical networks have been implicated in the etiopathogenesis of D/EE‐SWAS, largely because of their significant role in sleep‐related mechanisms and generation of interictal epileptiform discharges.^29^ This central role of the thalamus in D/EE‐SWAS pathogenesis is further supported by evidence of early thalamic lesions, which can be associated with the development of D/EE‐SWAS in over 80% of cases.^30^ Moreover, reductions in thalamic volume have been observed in patients with D/EE‐SWAS, even in the absence of macroscopic lesions,^31^ with additional evidence suggesting the presence of microarchitectural damage.^32^ Finally, a recent study provides direct evidence from human thalamocortical recordings that epileptic spikes in EE‐SWAS disrupt thalamic sleep spindle production, potentially compromising their function in memory consolidation.^33^ Disrupted activity and connectivity in the default mode network (DMN) has been also pointed out as a potential contributory factor to cognitive dysfunctions in individuals affected by D/EE‐SWAS, as already observed in various neuropsychiatric conditions including neurodevelopmental disorders. Building on previous positron emission tomography studies demonstrating metabolic changes in patients with D/EE‐SWAS,^23^ a distinctive functional fingerprint has indeed been identified, characterized by hypermetabolism in centroparietal regions, mainly colocalized with epileptic foci, and hypometabolism in DMN‐related structures, during both sleep and wakefulness.^34^ This metabolic pattern has consequently been linked to the potential remote inhibition of the DMN, with further corroboration by EEG‐fMRI studies.^24^ Ultimately, a complex network involving DMN structures has been demonstrated using coherence analysis applied to sleep EEG in patients with D/EE‐SWAS, irrespective of the aetiology and severity of epilepsy, with resolution after EEG renormalization.^35^ These central hubs appear to direct information flow to other regions, particularly the temporal cortex which has been implicated in the generation of epileptic activity based on advanced neurophysiological methods such as source reconstruction analysis.^24^, ^35^ This raises the question of whether the DMN drives changes in the epileptic network rather than the reverse.

Lastly, it would be of particular interest to explore the application of Menon's triple network model to D/EE‐SWAS. In this context, a recent resting‐state fMRI study on patients with D/EE‐SWAS has demonstrated reduced functional connectivity in both control executive network and salience network, providing a valuable conceptual framework that could serve as a foundation for future research.^36^

## FUTURE DIRECTIONS AND PRACTICAL CONSIDERATIONS

Future research on D/EE‐SWAS in a network‐based perspective should delve more deeply into the complex relationship between neurobiological and clinical measures.

Few studies have so far investigated correlations between neuropsychological features and functional neuroimaging data. Recent evidence has demonstrated a moderate yet significant correlation between Child Behaviour Checklist scores, but not IQ scores, and the total number of hypometabolic brain regions identified through fluorodesoxyglucose positron emission tomography‐computed tomography.^37^ In a more innovative network‐based approach, a resting state‐fMRI study reported altered variability in functional connectivity involving key salience network structures (i.e. the anterior cingulate cortex and anterior insula) which was associated with deficits in executive functions and attention.^38^

From a practical standpoint, advancing the understanding of D/EE‐SWAS necessitates the collection of data from a network‐based perspective across multiple domains, producing multimodal data sets within the same cohort of participants. Neuropsychological research data must be collected using standardized and shared protocols to ensure consistency and comparability across studies. To this end, a consensus has been proposed to develop a shared protocol of neurocognitive tests covering major neuropsychological domains. To advance beyond lesion‐based models and conventional neurodevelopmental syndromes, there is a crucial need for a refined, high‐granularity taxonomy for the description of neuropsychological profiles associated with epilepsy. The International Classification of Cognitive Disorders in Epilepsy based on the exploration of cognitive domains seems a promising tool for harmonization of the approach to cognitive diagnosis in adults.^39^ In line with this proposal, evaluation protocols for children should be based on a multidimensional cognitive evaluation encompassing, like in the adult model, the domains of language, memory, executive, visuospatial, and attention but also domains of critical relevance in the developmental age, including academic, social, and adaptive skills.

Concurrently, neuroimaging and neurophysiological research data should be gathered with a focus on connectomics to elucidate the complex neural connections and network disruptions associated with this condition. Along with more conventional quantitative EEG‐derived measures (spike‐wave index, slow‐wave downscaling), shared protocols should be implemented to gather comprehensive data on both structural and functional connectivity. This includes leveraging advanced MRI techniques (diffusion tensor imaging, resting‐state fMRI) to investigate the intrinsic functional networks and their potential alterations, and EEG coherence studies to identify global and local network disruptions that may underlie the pathophysiology of the disorder. Multimodal integration of these data, including simultaneous EEG‐fMRI studies, could offer a detailed understanding of the spatiotemporal characteristics of the epileptic networks involved. Given the transient and dynamic nature of the epileptiform activity in D/EE‐SWAS, it is also crucial to incorporate longitudinal neuroimaging and neurophysiological assessments, which would allow for the tracking of network changes over time.

Ultimately, genetic information is crucial to the study of D/EE‐SWAS because of the significant role it plays in shaping and determining the developmental trajectories of neural networks.

Moving forward, the integration of advanced computational methods has the potential to greatly enhance our ability to unravel the intricate interactions among the various dimensions of D/EE‐SWAS. Artificial intelligence and machine learning algorithms have already demonstrated utility in the study of neurodevelopmental and psychiatric disorders, as well as in epilepsy research.^40^ However, the efficacy of artificial intelligence tools in analysing extensive data sets hinges critically on the quality of the data incorporated into these models. It is imperative that standardized frameworks, built upon existing guidelines and best practices, are established to ensure the integrity of input data, thereby improving the generalizability and reliability of artificial intelligence‐driven findings.^41^ Moreover, in the context of rare epileptic syndromes, meaningful progress can only be achieved through collaborative efforts. These efforts must be underpinned by a shared conceptual framework, now provided by the International League Against Epilepsy definitions, and robust cross‐centre data collection protocols, which will facilitate the creation of large, harmonized data sets. Only through such initiatives can we develop a comprehensive understanding of these complex conditions, with the potential to guide the development of targeted interventions and the identification of biomarkers predictive of disease progression and treatment response.

## Data Availability

Data sharing not applicable to this article as no data sets were generated or analysed during the current study.
